# Fabrication of Perfluoropolyether Microfluidic Devices Using Laser Engraving for Uniform Droplet Production

**DOI:** 10.3390/mi15050599

**Published:** 2024-04-29

**Authors:** Eun Seo Kim, Mincheol Cho, Inseong Choi, Sung-Wook Choi

**Affiliations:** Department of Biotechnology, Biomedical and Chemical Engineering, The Catholic University of Korea, 43 Jibong-ro, Wonmi-gu, Bucheon-si 14662, Gyeonggi-do, Republic of Korea; aog1313@naver.com (E.S.K.); mincheol724@catholic.ac.kr (M.C.); ins94innn@daum.net (I.C.)

**Keywords:** perfluoropolyether, microfluidics, laser engraving, droplet

## Abstract

A perfluoropolyether (PFPE)-based microfluidic device with cross-junction microchannels was fabricated with the purpose of producing uniform droplets. The microchannels were developed using CO_2_ laser engraving. PFPE was chosen as the main material because of its excellent solvent resistance. Polyethylene glycol diacrylate (PEGDA) was mixed with PFPE to improve the hydrophilic properties of the inner surface of the microchannels. The microchannels of the polydimethylsiloxane microfluidic device had a blackened and rough surface after laser engraving. By contrast, the inner surface of the microchannels of the PFPE-PEGDA microfluidic device exhibited a smooth surface. The lower power and faster speed of the laser engraving resulted in the development of microchannels with smaller dimensions, less than 30 μm in depth. The PFPE and PFPE-PEGDA microfluidic devices were used to produce uniform water and oil droplets, respectively. We believe that such a PFPE-based microfluidic device with CO_2_-laser-engraved microchannels can be used as a microfluidic platform for applications in various fields, such as biological and chemical analysis, extraction, and synthesis.

## 1. Introduction

Microfluidic devices with microchannels have attracted considerable attention in a variety of scientific and industrial fields. Microfluidic devices can be used for the production of lipid nanoparticles and uniform droplets; the synthesis of chemical and biological molecules; the analyses of protein, DNA, and mRNA; and so on [1]. The key issues that must be considered in microfluidic devices are the properties of the device material; the pattern, dimension, and surface morphology of the microchannel; and the fabrication method. For example, the device material should be carefully selected in consideration of its hydrophilicity, chemical stability, mechanical properties, and non-specific adsorption of introduced ingredients for a specific application.

Monodisperse emulsion droplets have received considerable attention in many areas of research and industry. Emulsions can be characterized as a function of many factors, such as the properties of the dispersed and continuous phases, the volume fraction of the dispersed phase, the particle size distribution of the droplets, and the stability. There are three major types of emulsions: water-in-oil (W/O) emulsions, oil-in-water (O/W) emulsions, and complex emulsions such as water-in-oil-in-water (W/O/W) emulsions [2].

Droplet-based microfluidics has a wide range of applications in biomedicine, including particle synthesis, biomolecule analysis, cell biology, diagnostics, drug delivery, and drug screening. In biological studies, for example, droplet-based microfluidic devices have been used to extract DNA [3] and to perform DNA sequencing [4]. Zubaite et al., used droplet-based microfluidic systems for the separation, amplification, and condensation of single DNA molecules from 3 pL volume droplets and converted them into DNA–magnesium–inorganic pyrophosphate particles ~1300 nm in size [5]. In addition, in the pharmaceutical industry, it is essential to design drug delivery systems with small droplet size and narrow size distribution to avoid variability in drug release behavior and to prevent the accumulation of microspheres at undesirable sites in the body [6].

The microfluidic emulsification strategy enables the production of emulsions with low polydispersity (less than 5%) in a highly reproducible manner [7,8]. A narrow size distribution is also desirable as it mitigates Ostwald ripening by reducing the effective La-place pressure difference that induces mass transfer [9]. It can provide 100% encapsulation efficiency and ensure the precise and independent control of droplet size and droplet production rate [10]. In addition, different functional ingredients can be added to the droplets independently and individually during or after the droplet generation [11,12,13]. The generation of monodisperse emulsions with small microfluidic devices has led to many studies in different fields and many excellent reviews, including food applications [11].

A variety of materials have been used for the fabrication of microfluidic devices, including polydimethylsiloxane (PDMS), organic polymers (e.g., polystyrene), silicon wafer, and glass. PDMS and organic polymers have been widely used in microfluidic devices due to their optical transparency, biocompatibility, low cost, and ease of fabrication [14].

Oil droplet generation is key to several microfluidic applications, including the synthesis of advanced nano- and micro-materials in oil droplet microreactors. O/W emulsions are widely used in food processing [15], pharmaceutical or clinical production [16], material production [17], cosmetics [18], the petroleum industry [19], etc. O/W emulsions usually require the use of organic solvents capable of dissolving polymers [20]. However, the high hydrophobicity and swelling properties of organic solvents of PDMS and organic polymers have limited their expanded use [21]. Therefore, it is not easy to produce O/W emulsions with microfluidic devices using these materials.

In response, there have been efforts to modify the surface of PDMS to make it hydrophilic. Oxidation of the polymer surface using plasma or ultraviolet (UV) irradiation is a well-established approach to produce hydrophilic PDMS surfaces. However, as the uncured hydrophobic polymer chain migrates to the surface, this effect is temporary and the hydrophobic properties of PDMS return after a few minutes of plasma or UV exposure [22]. In addition, the main drawbacks of silicon wafer or glass microfluidic devices are the complexity and cost involved in manufacturing.

Therefore, a perfluoropolyether (PFPE) elastomer has recently emerged as an alternative material in microfluidic devices due to its attractive properties, such as its low surface energy and toxicity, along with well its high chemical and solvent resistance rates [23]. The low surface energy of PFPE is imparted by fluorination, and the difluoromethylene (-CF_2_-), difluoromethyl (-CF_2_H), and trifluoromethyl (-CF_3_) groups were found to exhibit surface energies of ~18 mN/m, ~15 mN/m, and ~6 mN/m, respectively [24]. In addition, the maximum swelling ratio of 3D-printed PFPE methacrylate was reported to be about 14% in tetrahydrofuran and about 11% in dichloromethane, which is 10-fold lower compared to PDMS [25]. The PFPE elastomer can be crosslinked by the addition of photoinitiator under UV light, thus suggesting its excellent processability. Despite the above advantages, PFPE still has limitations in its various applications, particularly in the production of oil-in-water emulsions, due to its hydrophobic properties [26].

The methods that can be used in the fabrication of microfluidic devices generally include injection molding [27], replica molding, three-dimensional (3D) printing [28], soft lithography [29], micromachining (e.g., laser engraving), and some combination of these techniques. Several researchers have fabricated microfluidic devices with micro- or nanochannels using these methods. Li et al., demonstrated this microfluidic approach through injection molding for single-cell analysis, which can organize up to 10,000 single cells [30]. Jans et al., developed a 3D-printed parallelized microfluidic device platform for the fabrication of dual emulsion and microgel capsules [31]. Bartlett et al., fabricated round-shape microchannels using soft lithography techniques [32]. Soft lithography technology is based on optical projection printing system technology and is one of the key processes for processing and fabricating microchannels. It is a technology that reduces the image on the mask using a high-aperture lens system and projects it onto a photoresist-coated substrate to engrave patterns on the surface of the substrate [33]. It is commonly used to fabricate microfluidic devices because of its high reproducibility and accuracy. Despite these advantages, the method requires a complicated process and expensive fabrication equipment [34].

However, the laser engraving technique has the notable advantages of high resolution and precision, non-contact qualities, and high-speed fabrication [35,36]. Moreover, laser engraving machines are commercially available and do not require high levels of user training for efficient operation [37]. Micropatterning using CO_2_ laser engraving is based on the photothermal effect. When the focused beam is focused onto the surface, the local temperature rapidly increases, and the materials melt and immediately evaporate, ultimately creating microchannels [38]. The laser beam rapidly raises the temperature of the small area on the surface of the material due to the pulsed nature and small spot size [39]. Therefore, CO_2_ laser engraving is more suitable for the processing of materials with low thermal conductivity (non-metals), such as polymers like polymethylmethacrylate (PMMA) [40]. For example, Yang et al., developed a PMMA-based microfluidic device for the encapsulation of ampicillin in chitosan microparticles using CO_2_ laser engraving [41]. Nayak et al., evaluated the effects of CO_2_ laser power and velocity on surface profiles, such as the depth and width of the microchannels of PMMA substrates [42]. Yuan et al., carried out experimental and theoretical investigations on the processing of PMMA substrates using CO_2_ laser engraving [43]. However, when organic solvents diffuse into PMMA, PMMA expands, causing local stress or deformation and cracking due to the diffusion-induced movement of PMMA chains. Therefore, PMMA is not suitable for organic solvents and its use in microfluidic devices is difficult [44]. In addition, the surface achieved on CO_2_-laser-engraved PMMA microchannels is rough. Roughness typically ranges in a few microns [45,46,47]. Initially, the influence of the CO_2_ laser parameters on the microchannel dimensions and the laser–PMMA interaction theory received much attention [48]. The increase in surface roughness can be easily explained by the increased formation of polymer residues due to variations in convective material ejection. This is exacerbated at higher power, due to raster scanning and the Gaussian power distribution of the laser. As the power is increased to achieve greater engraving depth, the size of the resulting grooves increases. This effect could be minimized using solvent vapor polymer reflow cleaning [49].

In addition, most polymers leave contamination and soot when exposed to CO_2_ lasers, so only some polymers are suitable for CO_2_ laser engravings. For example, polycarbonate leaves a brown mark when exposed to CO_2_ lasers [50]. As a result, CO_2_ lasers have the disadvantage of being limited to the materials that are available.

The aim of this study, which overcomes these limitations, is to produce microfluidic chips using hydrophilic-modified UV-cured PFPE polymers with high thermal stability in a simple way using CO_2_ laser engraving. Also, hydrophilic modification is easily achieved by UV curing after mixing a fluorinated polymer with a hydrophilic polymer [51].

In this work, we used CO_2_ laser engraving to fabricate a PFPE-based microfluidic device with cross-junction microchannels. Polyethylene glycol diacrylate (PEGDA), a hydrophilic macromonomer, was added to increase the hydrophilicity of the inner surface of the microchannels. The dimensions (width and depth) of the microchannels were controlled by adjusting the power and speed of the laser. We demonstrated the production of uniform water or oil droplets using PFPE and PFPE-PEGDA microfluidic devices, respectively. To our knowledge, this is the first demonstration of a PFPE-based microfluidic device micropatterned using a CO_2_ laser.

## 2. Materials and Methods

### 2.1. Materials

PDMS elastomers and curing agents (Sylgard^®^ 184) were purchased from Sewang Hitech (Gimpo, Republic of Korea). PFPE (Fluorolink MD 700, Solvay, Brussels, Belgium), PEG diacrylate (PEG-DA, Mn = 575, Sigma-Aldrich, St. Louis, MO, USA), and 2-hydroxy-2-methylpro-piophenone (Darocur 1173, Sigma-Aldrich) were used to fabricate the microfluidic device. Poly(vinyl alcohol) (PVA, Aldrich) was used as the surfactant for the continuous water phase. Isododecane (Alfa Aesar, Haverhill, MA, USA) was used for the oil phase.

### 2.2. Laser Engraving onto PDMS and PFPE-PEGDA Slabs

A PDMS mixture (PDMS elastomers and curing agents in a 10:1 weight ratio) was poured into a petri dish. After degassing for 30 min, the petri dish was placed in an oven at a temperature of 60 °C for 5 h for curing, after which a PDMS slab was obtained. A mixture of PFPE and a photoinitiator with or without PEGDA (20 wt.% against PFPE) was poured between two glass plates containing a spacer (0.5 mm thickness) and then exposed to UV light for 100 s using UV curing equipment (Innocure 850, Lichtzen, Gunpo-si, Republic of Korea). PFPE and PFPE-PEGDA20 slabs were obtained after washing with ethanol.

The obtained PDMS and PFPE-PEGDA slabs were engraved using a CO_2_ laser (40W, ML4040, Machine Shop, Paju-si, Republic of Korea). The laser power ranged from 6.4 to 9.1% at a speed of 1.0 mm/s, whereas the laser speed ranged from 0.5 to 5.0 mm/s at a power of 7.0%. After laser engraving, the inner surface morphology of the micropattern was observed using a scanning electron microscope (SEM, S-4800, Hitachi, Tokyo, Japan). The width and depth of the micropatterns were measured from the SEM images using ImageJ software 1.54 (National Institutes of Health, Bethesda, MD, USA).

Atomic force microscopy (AFM, MultiMode E-8-AM, Bruker, Ettlingen, Germany) was used to evaluate the roughness of the engraved micropatterns in PDMS, PFPE, and PFPE-PEGDA20 slabs. The laser power and speed were adjusted to 7.0% and 5.0 mm/s, respectively. The scanning area was 5.0 μm × 5.0 μm and the scanning rate was 0.8 Hz. The mean surface roughness (R_a_) and root mean square roughness (R_q_) were measured using a Nanoscope (Veeco Instruments Inc., Plainview, NY, USA) (n = 5).

### 2.3. Fabrication of Microfluidic Devices

A microfluidic pattern was designed using Adobe Illustrator software 2020 (Adobe Systems, San Jose, CA, USA). The microfluidic device consisted of one PDMS layer and two PFPE-based layers. The top PDMS layer had three holes for the inlet and outlet. The second layer was a PFPE slab with three holes matched to the top PDMS layer. The third PFPE-based layer had microchannels that were engraved using the CO_2_ laser at 6.7% power and a 1.0 mm/s speed. The CO_2_ laser engraving for the cross-junction was conducted at 6.4% power and a 1.0 mm/s speed to minimize the dimensions of the microchannels. The laser speed and power for all holes were 1.0 mm/s and 11.0%, respectively. The three layers were combined and fixed between the two acrylic plates using screws. The inlet and outlet holes were connected to the syringe pumps (KDS 100, KD Scientific, Boston, MA, USA) using Tygon^®^ tubes (1/32 inch I.D. × 1/16 inch O.D.).

### 2.4. Production of Emulsion Droplets

For the oil-in-water emulsion, isododecane and an aqueous PVA solution (2 wt.%) were used as the discontinuous and continuous phases, respectively, in the PFPE-PEG microfluidic device. Meanwhile, for the water-in-oil emulsion, water and isododecane with Span 80 (2 wt.%) were used as the discontinuous and continuous phases, respectively, in the PFPE microfluidic device. The flow rate of the continuous phase was varied from 1.0 to 8.0 mL/h at 0.2 mL/h of the flow rate of the discontinuous phase. The emulsion droplet formation was observed using a high-speed camera (CR3000x2, Optronis, Kehl, Germany) to obtain time-lapse images. The produced droplets were collected on a concave glass and observed through optical microscopy (BX-43, Olympus, Tokyo, Japan). The average sizes and standard deviations of the droplets were calculated using ImageJ (National Institutes of Health, Bethesda, MD, USA) (n = 100). The coefficient of variation (CV) was calculated by dividing the standard deviation by the average value.

## 3. Results and Discussion

### 3.1. Fabrication of the Microfluidic Device

**Scheme 1:**
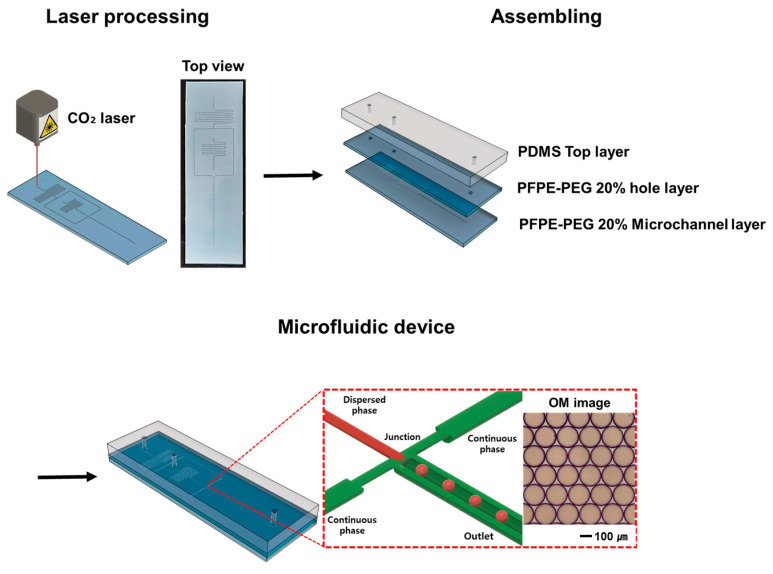
Development of microchannels using the CO_2_ laser engraving and fabrication of microfluidic devices for droplet generation.

Scheme 1 presents schematic illustrations of the laser engraving and the fabrication of the PFPE-based microfluidic device for uniform droplet generation. The microfluidic device consisted of three layers: a PDMS top layer with inlet/outlet holes, a second PFPE-based layer with inlet/outlet holes, and a third PFPE-based layer with microchannels engraved by a CO_2_ laser. There were two inlets for the introduction of continuous and discontinuous phase and an outlet for the outflow of the resulting emulsion droplets. The continuous phase introduced through the inlet was divided into two flow streams. The discontinuous phase was in contact with the continuous phase at the cross-junction and grew over time, pinching off to form emulsion droplets [52].

### 3.2. Surface Roughness of Engraved Microchannel

**Figure 1:**
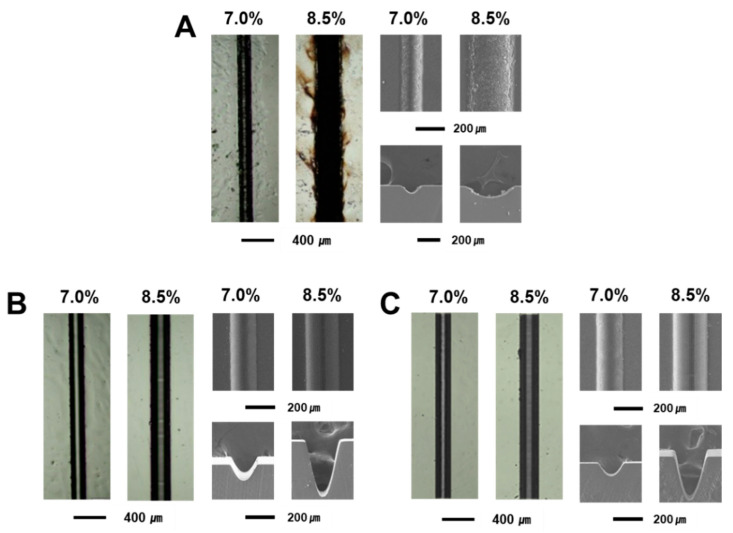
Optical microscopy and SEM images of microchannel morphology at different laser power rates (7.0 and 8.5%) and laser speed of 1.0 mm/s for (**A**) PDMS, (**B**) PFPE, and (**C**) PFPE-PEGDA20 slabs. Scale bars are 200 μm and 400 μm.

Figure 1 shows the optical microscopy and SEM images of the PDMS, PFPE, and PFPE-PEGDA20 microchannels engraved at the different power rates of 7.0 and 8.5%. PEGDA Mn 575 exhibited more miscibility to PFPE and stronger mechanical properties. As shown in Figure 1A, the PDMS microchannel engraved at 7.0 and 8.5% power rates exhibited a scorched and rough surface. It is known that the thermal defects of the scorching, shrinkage, and re-solidification of PDMS after laser ablation are prone to form on the surface [53,54]. Moreover, the depth of the engraved microchannel was relatively low. By contrast, the surfaces of PFPE-based microchannels remained unburnt and smooth (Figure 1B,C). The depths of the PFPE and PFPE-PEGDA20 microchannels were greater than the depth of the PDMS microchannel due to the thermal stability of PFPE [55,56]. The cross-section of the microchannels can have the shape of a triangle or a semicircle. The rectangle cross-section cannot be fabricated with this laser engraving method.

The surface roughness of the microchannels affects the fluid flow characteristics and adsorption of the introduced molecules [57,58]. A smoother surface of the microchannel might generally be preferable in most microfluidic devices. Therefore, AFM analysis was conducted to quantitatively evaluate the surface roughness of the engraved microchannels.

**Figure 2:**
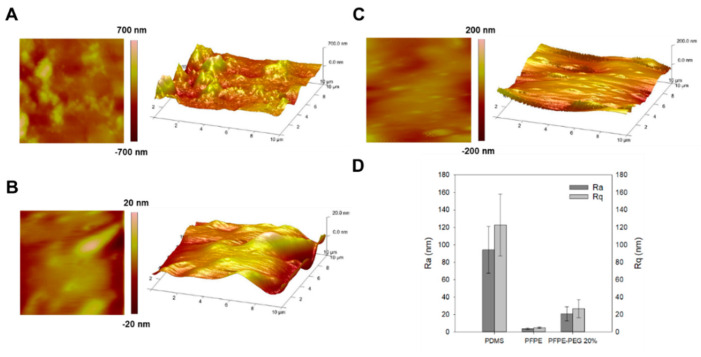
Atomic force microscopy images of the laser-engraved microchannels for (**A**) PDMS, (**B**) PFPE, and (**C**) PFPE-PEGDA20 slabs. (**D**) Quantified surface roughness of average surface roughness (R_a_) and root-mean-square roughness (R_q_) of three different slabs.

Figure 2A–C show topographic images of the PDMS, PFPE, and PFPE-PEGDA20 microchannels, respectively. A brighter color represents an area with higher height in the AFM topography images. The PDMS microchannel exhibited many sharp peaks and valleys, thus suggesting a rough surface. Meanwhile, the PFPE-based microchannels showed relatively smooth surface morphology. Figure 2D shows the quantified R_a_ and R_q_ values of the three different microchannels. The R_a_ (94.06 ± 26.83 nm) and R_q_ (122.7 ± 35.69 nm) values of the PDMS microchannel were much higher than those of the PFPE and PFPE-PEGDA20 microchannels. The PFPE microchannel (R_a_ 3.65 ± 0.73 nm and R_q_ 4.78 ± 0.97 nm) exhibited a smoother surface than the PFPE-PEGDA20 microchannels (R_a_ 20.78 ± 8.26 nm and R_q_ 26.77 ± 10.49 nm). This can be attributed to the fact that PFPE exhibits high chemical and thermal stability due to the strong C-F and C-C bonding and shielding of the polymer backbone via the sheathing of the unpaired electrons of the fluorine atoms [59].

### 3.3. Dimension Control of Laser-Engraved Microchannel

The PDMS, PFPE, and PFPE-PEGDA20 slabs were engraved by varying the laser power and speed, and then the dimensions of the microchannels were examined in terms of width and depth. To observe the effect of laser power, it was varied from 6.5 to 9.0% at a laser speed of 1.0 mm/s. Meanwhile, to observe the effect of laser speed, it was adjusted from 1.0 to 6.0 mm/s at a laser power rate of 7.0%. In all the slabs, a higher laser power resulted in higher energy transfer and more heat, which increased the width and depth of the microchannel [60,61]. As the laser speed increased, the energy transfer per unit length decreased, thus resulting in reductions in both the width and depth of the microchannel [60].

**Figure 3:**
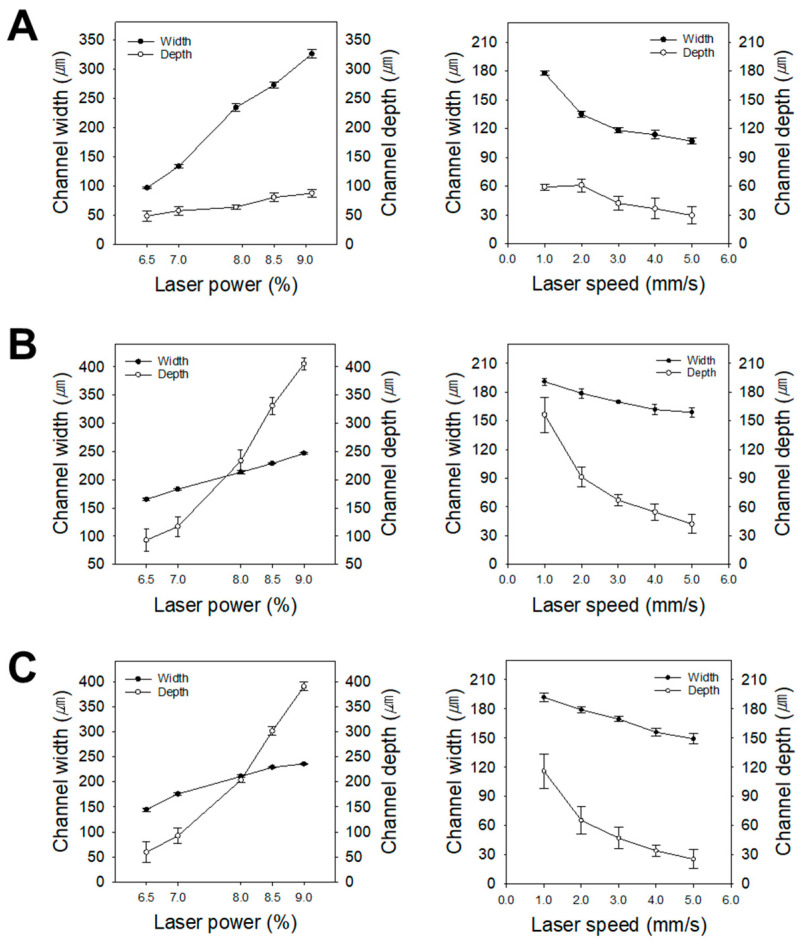
Variations in microchannel width and depth at different laser power rates and speeds for (**A**) PDMS, (**B**) PFPE, and (**C**) PFPE-PEGDA20 slabs.

By changing the laser power and speed, the microchannel width and depth of the PDMS slab could be controlled in the ranges of 96.78 ± 1.74 to 326.03 ± 6.89 μm (for the range of width depending on the power), 48.19 ± 8.27 to 87.23 ± 6.49 μm (for the range of depth depending on the power), 106.80 ± 3.04 to 214.18 ± 9.70 μm (for the range of width depending on speed), and 29.48 ± 9.00 to 59.04 ± 3.15 μm (for the range of depth depending on speed), as shown in Figure 3A. The width and depth of the microchannels of the PFPE slabs were in the range of 147.51 ± 4.56 to 246.77 ± 1.72 μm (for the range of width depending on the power), 83.32 ± 33.77 to 405.31 ± 10.16 μm (for the range of depth depending on the power), 158.87 ± 4.60 to 201.91 ± 2.75 μm (for the range of width depending on speed), and 42.20 ± 9.94 to 265.54 ± 31.39 μm (for the range of depth depending on speed), as shown in Figure 3B. For the PFPE-PEGDA20 slab, the width and depth were controlled from 115.09 ± 5.32 to 235.90 ± 0.87 μm (for the range of width depending on the power), from 44.30 ± 24.98 to 390.23 ± 8.82 μm (for the range of depth depending on the power), from 149.25 ± 5.22 to 205.47 ± 8.75 μm (for the range of width depending on speed), and from 25.53 ± 9.55 to 192.79 ± 41.20 μm (for the range of depth depending on speed), as shown in Figure 3C.

Relative to the depth, the width of the microchannel in the PDMS slab was found to be highly dependent on the laser power and speed [62]. However, the depth of the microchannels in the PFPE-based slabs was substantially affected by the laser power and speed. This tendency might also be attributable to the thermal stability of PFPE. These results confirm that the dimensions of the microchannels can be easily adjusted based on the laser conditions.

### 3.4. Production of Emulsion Droplets

By utilizing the effect of the laser condition on the microchannel dimension, the PFPE and PFPE-PEGDA20 microfluidic devices were fabricated for the purpose of generating uniform emulsion droplets. The laser power and speed were determined appropriately for the inlet and outlet microchannels and cross-junction of the microfluidic device. The width and depth of the inlet and outlet microchannels fabricated at the conditions of 7.3% and 4.10 mm/s were 203.19 and 214.82 μm, respectively. In general, the size of the generated droplet was affected by the microchannel dimension of the cross-junction. Therefore, the laser power and speed for the cross-junction were set to 6.4% and 1.0 mm/s, respectively, to fabricate microchannels with small widths and depths.

**Figure 4:**
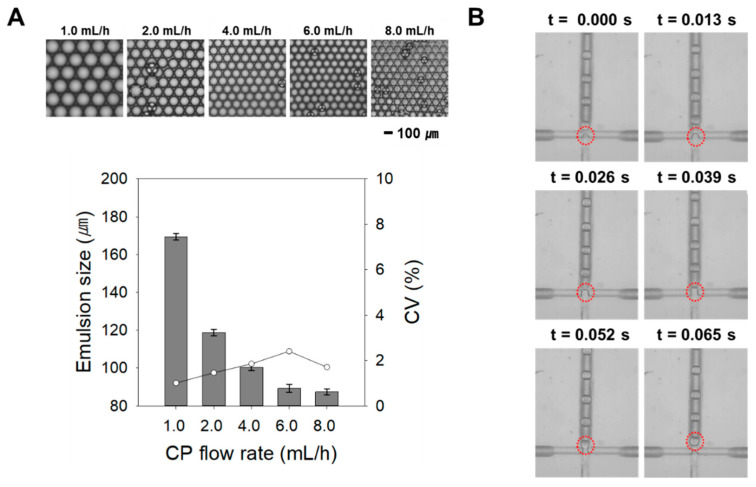
(**A**) Size and CV variations in water droplets with respect to the flow rate of the continuous phase in the PFPE microfluidic device and (**B**) representative time-lapse images at the cross-junction (1 mL/h of the continuous-phase flow rate).

In general, the hydrophobic and hydrophilic microchannels were found to be suitable for the generation of the W/O and O/W emulsion system, respectively. Therefore, the PFPE and PFPE-PEGDA20 microfluidic devices were successfully used for the continuous generation of the water and oil droplets, respectively. Figure 4A shows optical microscopy images and the variation in the sizes of water droplets generated in the PFPE microfluidic device. The flow rate of the continuous phase was varied from 1.0 to 8.0 mL/h. An increase in the flow rate of the continuous phase led to a decrease in droplet size from 169.43 ± 1.71 μm to 87.46 ± 1.49 μm, while the CV values remained under 3%, therefore suggesting high uniformity. Figure 4B shows time-lapse images of water droplet formation taken by the high-speed camera. One emulsion droplet was produced every 0.065 s at the PFPE cross-junction.

**Figure 5:**
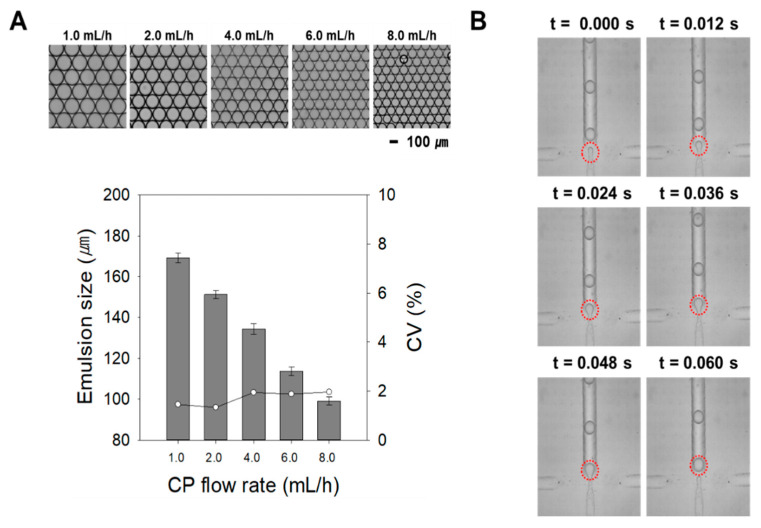
(**A**) Size and CV variations in oil droplets with respect to the flow rate of the continuous phase in the PFPE-PEGDA20 microfluidic device and (**B**) representative time-lapse images at the cross-junction (1 mL/h of the continuous-phase flow rate).

Figure 5A shows optical microscopy images and variations in the sizes of the oil droplets that were formed in the PFPE-PEGDA20 microfluidic device. An increase in the flow rate of the continuous phase from 1.0 to 8.0 mL/h led to a decrease in droplet size from 169.24 ± 2.45 μm to 99.07 ± 1.94 μm, while the CV was kept at less than 2%. The time-lapse images revealed that one emulsion droplet was produced every 0.060 s in the PFPE-PEGDA20 microfluidic device (Figure 5B).

## 4. Conclusions

We successfully fabricated a PFPE-based microfluidic device using a CO_2_ laser and demonstrated the continuous production of the uniform water or oil droplets. The CO_2_ laser engraving method can be a flexible, fast, low-cost, and versatile technique for the rapid prototyping of microchannels of PFPE-based microfluidic devices. The main features of our approach are as follows: (1) the fabrication of microchannels by employing laser engraving, (2) the creation of hydrophilic microchannels by adding PEGDA to PFPE, and (3) the production of monodisperse water or oil droplets with tunable sizes. Laser processing parameters (such as power and speed) showed a relationship with microchannel dimension. The dimensions of the microchannels can be easily controlled by adjusting the laser processing parameters. Both the sizes of the water and oil droplets were controlled to less than 100 μm by changing the dimensions of the cross-junction. We believe that the use of PFPE-based microfluidic devices can overcome the drawbacks of PDMS-based microfluidic devices. Beyond emulsion production, our next goals are focused on the fabrication of uniform microspheres using organic solvents for various applications, such as in the pharmaceutical, food, and cosmetic industries.

## Data Availability

Data are contained within the article.

## References

[1] Ahn G.Y., Choi I., Song M., Han S.K., Choi K., Ryu Y.H., Oh D.H., Kang H.W., Choi S.W. (2022). Fabrication of Microfiber-Templated Microfluidic Chips with Microfibrous Channels for High throughput and Continuous Production of Nanoscale Droplets. ACS Macro Lett..

[2] Nour A.H. (2018). Emulsion types, stability mechanisms and rheology: A review. Int. J. Innov. Res. Sci. Stud. (IJIRSS).

[3] Zhang Y., Park S., Yang S., Wang T.H. (2010). An all-in-one microfluidic device for parallel DNA extraction and gene analysis. Biomed. Microdevices.

[4] Abate A.R., Hung T., Sperling R.A., Mary P., Rotem A., Agresti J.J., Weiner M.A., Weitz D.A. (2013). DNA sequence analysis with droplet-based microfluidics. Lab Chip.

[5] Zubaite G., Simutis K., Galinis R., Milkus V., Kiseliovas V., Mazutis L. (2017). Droplet microfluidics approach for single-DNA molecule amplification and condensation into DNA-magnesium-pyrophosphate particles. Micromachines.

[6] Ho T.M., Razzaghi A., Ramachandran A., Mikkonen K.S. (2022). Emulsion characterization via microfluidic devices: A review on interfacial tension and stability to coalescence. Adv. Colloid Interface Sci..

[7] Rosen M.J., Kunjappu J.T. (2012). Surfactants and Interfacial Phenomena.

[8] Kruglyakov P.M. (2000). Hydrophile-Lipophile Balance of Surfactants and Solid Particles: Physicochemical Aspects and Applications.

[9] Sugiura S., Nakajima M., Kumazawa N., Iwamoto S., Seki M. (2002). Characterization of spontaneous transformation-based droplet formation during microchannel emulsification. J. Phys. Chem. B.

[10] Romanowsky M.B., Abate A.R., Rotem A., Holtze C., Weitz D.A. (2012). High throughput production of single core double emulsions in a parallelized microfluidic device. Lab Chip.

[11] Vladisavljević G.T., Al Nuumani R., Nabavi S.A. (2017). Microfluidic production of multiple emulsions. Micromachines.

[12] Chu L.Y., Utada A.S., Shah R.K., Kim J.W., Weitz D.A. (2007). Controllable monodisperse multiple emulsions. Angew. Chem. Int. Ed..

[13] Zhang M., Wang W., Xie R., Ju X., Liu Z., Jiang L., Chen Q., Chu L. (2015). Controllable microfluidic strategies for fabricating microparticles using emulsions as templates. Particuology.

[14] Lyer M.A., Eddington D.T. (2019). Storing and releasing rhodamine as a model hydrophobic compound in polydimethylsiloxane microfluidic devices. Lab Chip.

[15] Garti N. (1997). Progress in Stabilization and Transport Phenomena of Double Emulsions in Food Applications. LWT-Food Sci. Technol..

[16] Okochi H., Nakano M. (2000). Preparation and evaluation of w/o/w type emulsions containing vancomycin. Adv. Drug Deliv. Rev..

[17] Oh C., Chung S.C., Shin S.I., Kim Y.C., Im S.S., Oh S.G. (2002). Distribution of macropores in silica particles prepared by using multiple emulsions. J. Colloid Interface Sci..

[18] Lee J.S., Kim J.W., Han S.H., Chang I.S., Kang H.H., Lee O.S., Oh S.G., Suh K.D. (2004). The stabilization of L-ascorbic acid in aqueous solution and water-in-oil-in-water double emulsion by controlling pH and electrolyte concentration. J. Cosmet. Sci..

[19] Schramm L.L. (1992). Fundamentals and applications in the petroleum Industry. Adv. Chem..

[20] Belletti G., Buoso S., Ricci L., Guillem-Ortiz A., Aragón-Gutiérrez A., Bortolini O., Bertoldo M. (2021). Preparations of poly (lactic acid) dispersions in water for coating applications. Polymers.

[21] Gokaltun A., Yarmush M.L., Asatekin A., Usta O.B. (2017). Recent advances in nonbiofouling PDMS surface modification strategies applicable to microfluidic technology. Technology.

[22] Trantidou T., Elani Y., Parsons E., Ces O. (2017). Hydrophilic surface modification of PDMS for droplet microfluidics using a simple, quick, and robust method via PVA deposition. Microsyst. Nanoeng..

[23] Yang Y.W., Hentschel J., Chen Y.C., Lazari M., Zeng H., van Dam R.M., Guan Z. (2012). Clicked fluoropolymer elastomers as robust materials for potential microfluidic device applications. J. Mater. Chem..

[24] Jarvis N.L., Zisman W.A. (1965). Surface chemistry of fluorochemicals. Kirk-Othmer Encycl. Chem. Technol..

[25] Risch P., Kotz F., Helmer D., Rapp B.E. (2019). 3D printing of highly fluorinated methacrylates for the rapid prototyping of transparent and chemically-resistant microfluidic devices. Microfluidics, BioMEMS, and Medical Microsystems XVII, San Francisco, CA, USA, 2–7 February 2019.

[26] Jeong H.H., Han S.H., Yadavali S., Kim J., Issadore D., Lee D. (2018). Moldable Perfluoropolyether–Polyethylene Glycol Networks with Tunable Wettability and Solvent Resistance for Rapid Prototyping of Droplet Microfluidics. Chem. Mater..

[27] Kim Y., Song J., Lee Y., Cho S., Kim S., Lee S.R., Park S., Shin Y., Jeon N.L. (2021). High-throughput injection molded microfluidic device for single-cell analysis of spatiotemporal dynamics. Lab Chip.

[28] Metha V., Rath S.N. (2021). 3D printed microfluidic devices: A review focused on four fundamental manufacturing approaches and implications on the field of healthcare. Bio-Des. Manuf..

[29] Paoli R., Di Giuseppe D., Badiola-Mateos M., Martinelli E., Lopez-Martinez M.J., Samitier J. (2021). Rapid manufacturing of multilayered microfluidic devices for organ on a chip applications. Sensors.

[30] Li Y., Motschman J.D., Kelly S.T., Yellen B.B. (2020). Injection molded microfluidics for establishing high-density single cell arrays in an open hydrogel format. Anal. Chem..

[31] Jans A., Lölsberg J., Omidinia-Anarkoli A., Viermann R., Möller M., De Laporte L., Wessling M., Kuehne A.J. (2019). High-throughput production of micrometer sized double emulsions and microgel capsules in parallelized 3D printed microfluidic devices. Polymers.

[32] Bartlett N.W., Wood R.J. (2016). Comparative analysis of fabrication methods for achieving rounded microchannels in PDMS. J. Micromech. Microeng..

[33] Han X., Zhang Y., Tian J., Wu T., Li Z., Xing F., Fu S. (2022). Polymer-based microfluidic devices: A comprehensive review on preparation and applications. Polym. Eng. Sci..

[34] Ahn G.Y., Choi I., Song M., Han S.K., Choi K., Choi S.W. (2021). Production of Uniform Microspheres Using a Simple Microfluidic Device with Silica Capillary. Macromol. Res..

[35] Mohammed M.I., Quayle K., Alexander R., Doeven E., Nai R., Haswell S.J., Kouzani A.Z., Gibson I. (2015). Improved manufacturing quality and bonding of laser machined microfluidic systems. Procedia Technol..

[36] Thomas T., Agrawal A. (2023). Design and fabrication of microfluidic devices: A cost-effective approach for high throughput production. J. Micromech. Microeng..

[37] Vargas M.J., Nieuwoudt M., Yong R.M., Vanholsbeeck F., Williams D.E., Simpson M.C. (2019). Excellent quality microchannels for rapid microdevice prototyping: Direct CO_2_ laser writing with efficient chemical postprocessing. Microfluid. Nanofluidics.

[38] Dudala S., Rao L.T., Dubey S.K., Javed A., Goel S. (2020). Experimental characterization to fabricate CO_2_ laser ablated PMMA microchannel with homogeneous surface. Mater. Today Proc..

[39] Guler M.T., Inal M., Bilican I. (2021). CO_2_ laser machining for microfluidics mold fabrication from PMMA with applications on viscoelastic focusing, electrospun nanofiber production, and droplet generation. J. Ind. Eng..

[40] Bilican I., Guler M.T. (2020). Assessment of PMMA and polystyrene based microfluidic chips fabricated using CO_2_ laser machining. Appl. Surf. Sci..

[41] Yang C.H., Huang K.S., Chang J.Y. (2007). Manufacturing monodisperse chitosan microparticles containing ampicillin using a microchannel chip. Biomed. Microdevices.

[42] Nayak N.C., Lam Y.C., Yue C.Y., Sinha A.T. (2008). CO_2_-laser micromachining of PMMA: The effect of polymer molecular weight. J. Micromech. Microeng..

[43] Yuan D., Das S. (2007). Experimental and theoretical analysis of direct-write laser micromachining of polymethyl methacrylate by CO_2_ laser ablation. J. Appl. Phys..

[44] Chuang Y.F., Wu H.C., Yang F., Yang T.J., Lee S. (2017). Cracking and healing in poly (methyl methacrylate): Effect of solvent. J. Polym. Res..

[45] Lawrence J., Li L. (2001). Modification of the wettability characteristics of polymethyl methacrylate (PMMA) by means of CO_2_, Nd: YAG, excimer and high power diode laser radiation. Mater. Sci. Eng. A.

[46] Jensen M.F., Noerholm M., Christensen L.H., Geschke O. (2003). Microstructure fabrication with a CO_2_ laser system: Characterization and fabrication of cavities produced by raster scanning of the laser beam. Lab Chip.

[47] Cheng J.Y., Wei C.W., Hsu K.H., Young T.H. (2004). Direct-write laser micromachining and universal surface modification of PMMA for device development. Sens. Actuators B Chem..

[48] Wang Z.K., Zheng H.Y., Lim R.Y.H., Wang Z.F., Lam Y.C. (2011). Improving surface smoothness of laser-fabricated microchannels for microfluidic application. J. Micromech. Microeng..

[49] Mohammed M.I., Alam M.N.H.Z., Kouzani A., Gibson I. (2016). Fabrication of microfluidic devices: Improvement of surface quality of CO_2_ laser machined poly (methylmethacrylate) polymer. J. Micromech. Microeng..

[50] Caiazzo F., Curcio F., Daurelio G., Minutolo F.M.C. (2005). Laser cutting of different polymeric plastics (PE, PP and PC) by a CO_2_ laser beam. J. Mater. Process. Technol..

[51] Wu J., Issadore D.A., Lee D. (2023). Patterning Wettability on Solvent-Resistant Elastomers with High Spatial Resolution for Replica Mold Fabrication of Droplet Microfluidics. ACS Appl. Mater. Interfaces.

[52] Moon S.K., Cheong I.W., Choi S.W. (2014). Effect of flow rates of the continuous phase on droplet size in dripping and jetting regimes in a simple fluidic device for coaxial flow. Colloids Surf. A Physicochem. Eng. Asp..

[53] Lin L., Chung C.K. (2021). PDMS microfabrication and design for microfluidics and sustainable energy application. Micromachines.

[54] Konari P.R., Clayton Y.D., Vaughan M.B., Khandaker M., Hossan M.R. (2021). Experimental analysis of laser micromachining of microchannels in common microfluidic substrates. Micromachines.

[55] Hu Z., Finlay J.A., Chen L., Betts D.E., Hillmyer M.A., Callow M.E., Callow J.A., DeSimone J.M. (2009). Photochemically cross-linked perfluoropolyether-based elastomers: Synthesis, physical characterization, and biofouling evaluation. Macromolecules.

[56] Hwang H.D., Kim H.J. (2011). UV-curable low surface energy fluorinated polycarbonate-based polyurethane dispersion. J. Colloid Interface Sci..

[57] Rechendorff K., Hovgaard M.B., Foss M., Zhdanov V.P., Besenbacher F. (2006). Enhancement of protein adsorption induced by surface roughness. Langmuir.

[58] Gamrat G., Favre-Marinet M., Le Person S., Baviere R., Ayela F. (2008). An experimental study and modelling of roughness effects on laminar flow in microchannels. J. Fluid Mech..

[59] Hu Z. (2009). Novel Perfluoropolyethers as Fouling-Release Coatings. Doctoral Dissertation.

[60] Prakash S., Kumar S. (2015). Fabrication of microchannels on transparent PMMA using CO_2_ Laser (10.6 μm) for microfluidic applications: An experimental investigation. Int. J. Precis. Eng. Manuf..

[61] Chen X., Li T., Shen J. (2016). CO_2_ laser ablation of microchannel on PMMA substrate for effective fabrication of microfluidic chips. Int. Polym. Process..

[62] Liu H.B., Gong H.Q. (2009). Templateless prototyping of polydimethylsiloxane microfluidic structures using a pulsed CO_2_ laser. J. Micromech. Microeng..

